# Mosquito and arbovirus surveillance in wetlands of South‐East England: Comparison of two adult mosquito traps, use of a novel trap with FTA™ cards and arbovirus testing

**DOI:** 10.1111/mve.70053

**Published:** 2026-02-06

**Authors:** Alexander G. C. Vaux, Harrison Hardy, Lucy Crossley, Colin J. Johnston, Anthony J. Abbott, Stephen Findlay‐Wilson, Amanda Callaghan, Jolyon M. Medlock

**Affiliations:** ^1^ Medical Entomology & Zoonoses Ecology, Centre for Climate and Health Security, UK Health Security Agency, Porton Down Salisbury UK; ^2^ School of Biological Sciences, University of Reading Reading UK; ^3^ Virology and Pathogenesis Group, UK Health Security Agency, Porton Down Salisbury UK

**Keywords:** mosquito, mosquito‐borne disease, surveillance, UK

## Abstract

Effective surveillance of mosquito populations is critical to monitoring and mitigating the spread of mosquito‐borne diseases (MBDs). This study evaluated the relative trapping efficiency of two widely used adult mosquito traps—the Biogents BG‐Sentinel (BGS) and the Mosquito Magnet® Executive (MM)—on British mosquitoes across four wetlands in south‐east England over a 12‐week period. A third trap, a Box‐Gravid trap fitted with an FTA™ card, was deployed to detect arboviruses such as West Nile virus (WNV) via saliva collection. A total of 11,584 adult female mosquitoes representing 15 species were collected. The MM trap captured a significantly higher total number of mosquitoes, while the BGS trap demonstrated greater species evenness and was significantly more effective at catching *Culex* (*Culex*) *pipiens* L., 1758. Spatial variation strongly influenced catch rates, with significant differences between wetlands. No evidence of WNV was detected in any mosquito pools or FTA™ cards. While both trap types yielded similar species richness, the MM trap may be optimal for collecting large sample sizes of mammalophagic species, whereas the BGS is better suited for capturing enzootic vectors such as *Culex pipiens s.l.*, and a broader spectrum of species. These findings provide evidence‐based recommendations for future UK wetland surveillance and enhance preparedness for emerging vector‐borne disease risks.

## Graphical abstract

- Trap performance: Mosquito Magnet® captured significantly more mosquitoes overall, while BG‐Sentinel showed greater species evenness and was more effective for *Culex pipiens s.l.* and broader species representation.
- Spatial variation: Mosquito abundance and species composition varied significantly between wetlands, highlighting the importance of site‐specific surveillance in wetland ecosystems.
- Surveillance outcomes: No West Nile virus detected; findings support complementary trap use to optimise sampling of mammalophagic and enzootic mosquito species in UK wetland monitoring programmes.

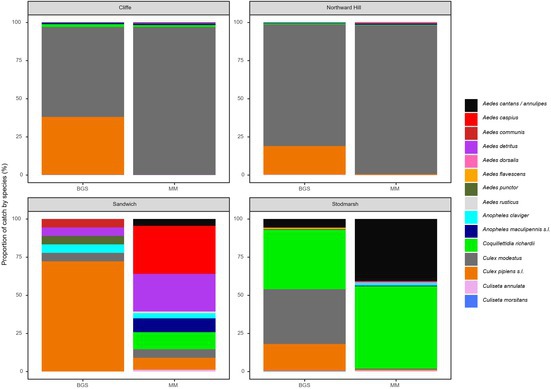

## INTRODUCTION

Mosquito‐borne pathogens and the diseases they cause are a public health issue globally, with millions of cases reported annually of diseases such as malaria, dengue, yellow fever, Zika and West Nile virus (WNV) (WHO, 2024), driven by manifold factors including habitat type and climate change (Franklinos et al., 2019). Mosquito‐borne diseases (MBDs) are driven by key vector species, and therefore, it is essential that bionomic data, especially their distribution and abundance, are available to inform control strategies for prevention or mitigation. Targeted collection of adult mosquitoes is most efficiently achieved through the use of adult mosquito traps, and these are an essential tool for entomologists to conduct surveillance and research (Service, 1976).

Adult female mosquitoes are primarily attracted to host‐seeking traps through the exploitation of the olfactory and, to a lesser extent, visual cues (Service, 1976) they utilise in natural host‐seeking behaviour. These cues mimic those emitted by potential hosts, such as humans or animals, enabling mosquitoes to locate bloodmeal sources required for egg production. Host‐seeking behaviour in mosquitoes is predominantly driven by olfactory signals, with carbon dioxide (CO_2_) being the principal long‐range attractant (Service, 1976; Takken, 1991). Female mosquitoes exhibit positive chemotaxis towards the source of an increasing CO_2_ concentration gradient and utilise this behaviour in host location via the chemosensation of exhaled CO_2_. Other volatiles, such as lactic acid, ammonia and compounds derived from the skin and its microbiome, act as short to medium range cues which attract host‐seeking females, and such compounds are utilised in synthetic lures to elicit similar responses (Cardé & Gibson, 2010; Coutinho‐Abreu et al., 2022; Dekker et al., 2001). These volatiles interact synergistically to enhance attraction, with specific blends eliciting strong responses in some species while being less effective for others (Takken & Verhulst, 2013).

Once within close proximity to a host, orientation towards a potential host is supported by visual cues; mosquitoes such as *Anopheles* (*Cellia*) *coluzzii* Coetzee & Wilkerson, 2013, detect contrasting shapes and movements, particularly in low‐light conditions when olfactory signals are present (Hawkes & Gibson, 2016). Contact with a host provides additional chemical information, such as skin‐secreted compounds and microbiome products, which further stimulate feeding. These gustatory cues are less explored but are likely critical for species‐specific host preferences (Zwiebel & Takken, 2004). The species‐specific responses of mosquitoes to host‐associated semiochemicals (Dormont et al., 2021) may, in part, explain the diversity of species captured by adult mosquito traps, which typically rely on different synthetic chemical lure blends. The range of use utilised by host‐seeking female mosquitoes, and their species‐specific effects, underscores the importance of tailored attractants and trap designs to optimise the surveillance and control of specific mosquito species (Bowen, 1991; Gillies, 1980). Further research into these complex mechanisms which modulate host‐seeking behaviour continues to refine our understanding of mosquito host‐seeking behaviour (Cardé, 2015; Dormont et al., 2021; Wooding et al., 2020). A range of mosquito traps designed to attract host‐seeking females are available including the Center for Disease Control and Prevention (CDC) miniature light trap, the Mosquito Magnet® Executive trap (MM), the Biogents BG‐Sentinel (BGS) and the Biogents BG‐Pro trap (Maciel‐de‐Freitas et al., 2006; Sudia & Chamberland, 1988).

In the United Kingdom, owing to concerns among land managers of trapping high numbers of moths in nature reserves, light traps are not habitually used, particularly in wetland studies (Medlock et al., 2024; Medlock & Vaux, 2015). In studies targeting invasive mosquitoes such as *Aedes* (*Stegomyia*) *albopictus* (Skuse, 1895), the BGS is considered the most effective trap when used with either BG‐Lure or BG‐Sweetscent, artificial scent lures (Akaratovic et al., 2017; Lühken et al., 2014), often in tandem with a standard ovitrap. When used with a CO_2_ source, the BGS may be suited to attracting a broader range of species (Lühken et al., 2014). However, the BGS requires frequent battery changes, typically every 3 days, for continued trap operation which is not conducive to weekly trap visits, and therefore, they have previously not been chosen for wetland studies in remote locations. MM traps are used extensively in the United Kingdom utilising an octenol lure, particularly in wetlands, and they have proved effective at attracting large numbers of mammalophagic female mosquitoes in a range of habitats (Medlock et al., 2024; Medlock & Vaux, 2015; Vaux et al., 2015, 2021, 2024). However, their operation requires the use of a propane or butane cylinder, for CO_2_ and heat generation, which may be undermined by logistical or equipment servicing issues preventing their deployment. In addition, in the United Kingdom, MM traps do not routinely trap ornithophagic species such as *Culex pipiens* typical biotype and therefore would not be suited for use in studies targeting this important enzootic vector (Vaux et al., 2021, 2024), particularly as part of arbovirus surveillance.

Arbovirus testing of trapped mosquitoes is an important component of MBD surveillance research in order to detect arboviruses within enzootic cycles and inform public health risk, and in recent years, this has provided insight into Usutu virus circulation (Lawson et al., 2022), and the first detection of WNV in mosquitoes trapped in the United Kingdom (Bruce et al., 2025; UKHSA, 2025). Novel methods of virus detection have included the modification of adult mosquito traps, particularly gravid traps to attract gravid females likely to have previously fed on a host, to house a FTA™ card within the trap for the purpose of collecting viral material from the deposited saliva of mosquitoes (Hall‐Mendelin et al., 2010). This approach to viral surveillance has been deployed across a number of studies, forming a key component of operational arbovirus surveillance across different geographic regions (Flies et al., 2015; Manzi et al., 2023; Wipf et al., 2019). This work signifies their first deployment for such purposes in UK wetlands.

This study aimed to evaluate the trapping efficiency of two commercially available and commonly used adult mosquito traps, specifically comparing the diversity of species caught and quantity of mosquitoes trapped by the BGS trap and the MM trap in four wetlands in South‐East England, in order to provide data to inform further studies in UK wetlands. Alongside this, the field testing of a novel combination of a bespoke Box‐Gravid trap and FTA™ cards was used to enhance arbovirus detection, particularly in species which oviposit in container habitats, such as *Cx. pipiens s.l*. All captured adult mosquitoes were tested for WNV as part of ongoing surveillance. Taken together these results inform surveillance strategies for putative mosquito vectors and arboviruses in UK wetland settings and contribute to preparedness for potential public risk in the future.

## METHODS

### Study locations and mosquito trapping

Mosquito traps were operated in four wetlands in Kent in south‐east England, targeting wetlands in the region where *Culex modestus*, a vector of WNV, is known to occur (Figure S1; Cliffe [latitude: 51.47364, longitude: 0.47749], Northward Hill [51.46209, 0.54479], Stodmarsh [51.30388, 1.18728] and Sandwich [51.27818, 1.36086]) (Vaux et al., 2024). Broadly, Cliffe and Northward Hill are dominated by grazing marsh including flooded grassland and vegetated permanent ditches bounding grassland grazed by livestock. Stodmarsh is a large nature reserve including wet woodland, grazing marsh, vegetated permanent ditches, wet grassland and reedbed. The Sandwich wetland is dominated by grazed arable fields, a large permanent ditch, and wet grassland, with noticeably less wetland habitat than the other wetlands studied here. This study did not quantify the scale of available habitat at each wetland. The traps were continuously operational across 11 weeks (calendar week 27 [5 July 2022] to week 38 [20 September 2022]). At each wetland, a Mosquito Magnet® (MM) Executive trap (Woodstream Corporation, St. Joseph, MO, USA), using a propane cylinder (13‐kg propane supplied by Calor Ltd., Warwick, UK) and baited with an octenol (1‐Octen‐3‐ol) lure (Woodstream Corporation) and a Biogents BG‐Sentinel (BGS) baited with BG‐Lure and CO_2_ (CO_2_ VB cylinder, 6.35 kg, supplied by BOC Ltd., Woking, UK). Traps were located at three locations (A, B, C) with over 50 metres between them, apart from at Sandwich where traps were 20 metres apart due to access restrictions. Each week, the MM and the BGS traps were rotated between locations, so that the trap previously at location A was moved to location B, and vice‐versa. The Box‐Gravid trap remained at location C for the duration of the study, as this trap was not part of the trap comparison element of this study.

The BGS traps were powered using a photovoltaic panel system, comprising a 12 V 15 Ah lead‐acid battery, 50 W photovoltaic panel and 10 A 12 V/24 V charge controller (Photonic Universe PU1024BW).

**FIGURE 1:**
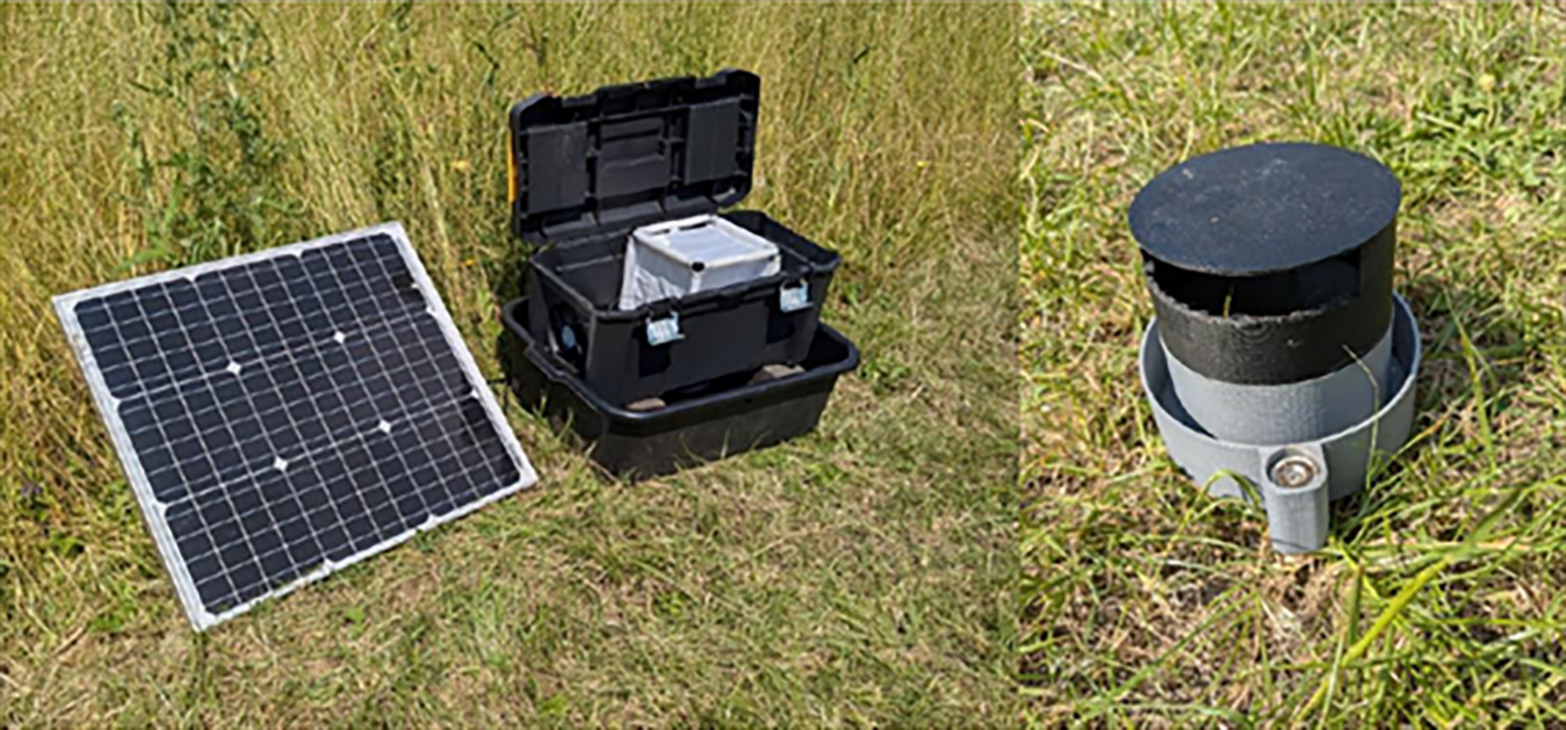
Box‐Gravid trap. Left: viewed with lid open, showing the BugDorm container housing trapped mosquitoes and the FTA™ card; outlet fan, photovoltaic panel and black water container are also shown. Right: 3D printed intake pipe, providing access for adult mosquitoes into the BugDorm and preventing mosquitoes falling back into the water.

Box‐Gravid traps (Figure 1) were based on the design of the modified Reiter‐Cummings gravid trap (Cummings, 1992; Fynmore et al., 2021; Reiter, 1983). These traps consisted of two main components: a lower tray with hay infusion (5 L of liquid attractant, consisting of a hay infusion made by mixing 0.9 kg of hay with 114 L of water) and the main trap body which held the components and BugDorm (model TM‐4M1515 Insect Rearing Cage [175 mm × 175 mm × 175 mm]). The lower tray was a stackable black plastic tray (470 × 350 × 170 mm) with a drainage hole, while the main trap body was a black toolbox (Stanley Tool Box 19″ 2 Pieces, 250 mm × 485 mm). The main body stood on two construction bricks placed within the tray. Two 60‐mm diameter holes were drilled centrally into the bottom and left‐hand side of the toolbox using a hole saw. The lower hole was used to insert a 3D‐printed intake pipe (Figure 1) and net holder with embedded magnets to affix it in place. The fan was bolted in place over the left‐hand side hole. The intake pipe was sealed at one end, with an exit hole on the side of the tube, to prevent mosquitoes from falling directly out of the trap and into the water. Inside the toolbox, a 120 mm 12 V DC axial fan, powered by the battery stowed within the toolbox, was bolted to the interior covering the left‐hand drilled hole. The fan was powered by a 12 V 15 aH lead‐acid battery connected to a charge controller and 50 W photovoltaic panel, allowing for unlimited run time. The BugDorm was placed over the entrance hole, oriented with the entrance port facing downwards in line with the intake pipe, and the entrance port sleeve was tucked into the sleeve holder on the 3D printed inlet, and the main trap body was then positioned over the lower tray.

Whatman™ FTA™ Classic cards (GE Healthcare Life Sciences, Buckinghamshire, UK) soaked in Manuka honey solution (10% honey to distilled water) were prepared using a procedure similar to previously published methods (Flies et al., 2015; Wipf et al., 2019). The cards were cut into equal quarters, soaked in honey solution overnight and placed into plastic resealable bags, with a 1.5 cm square hole cut in the front to provide mosquitoes access to the FTA™ cards. A cotton pad, soaked in the honey solution, was placed behind the FTA™ card to keep it moist during deployment. The FTA™ card was then affixed to the inside of the BugDorm™ on the opaque plastic side.

All traps were checked weekly on a Tuesday, and FTA™ cards, BugDorms and catch bags were removed and labelled and placed on dry ice. Upon return to the laboratory, mosquitoes were removed and stored at −80°C. FTA™ cards were stored at room temperature.

### Mosquito identification

Female mosquitoes were identified morphologically using taxonomic keys (Becker et al., 2010; Cranston et al., 1987; Snow, 1990). Where morphological identification was not possible, species were grouped as follows: *Cx. pipiens* sensu lato (*s.l*.) = *Cx*. (*Culex*) *pipiens* Linnaeus, 1758 and *Culex* (*Culex*) *torrentium* Martini, 1925; *Aedes cantans* /*annulipes* = *Ae*. (*Ochlerotatus*) *cantans* (Meigen, 1818) and *Ae*. (*Ochlerotatus*) *annulipes* (Meigen, 1830).

### RNA extraction and PCR

Mosquitoes were pooled into batches of ≤10 mosquitoes of the same species and transferred into Precellys tubes (Stretton Scientific, CK28‐R Cat. No.: P000916‐LYSKO‐A0). Then, 400 μL of RLT buffer (Indical Biosciences, UK), containing β‐mercaptoethanol and carrier RNA at 10 μL/mL was added to each tube and the samples processed using the Precellys tissue homogeniser (Bertin Technologies, Precellys 24) for 5 s at 4500 rpm for three cycles. An equal volume of 400‐μL molecular grade 70% ethanol (Fisher Scientific, UK) was added to each tube and mixed by inversion. Homogenate was passed through a QIAshredder (Qiagen, UK) at 13,000 rpm for 2 min. Total RNA was extracted using the Bio‐Sprint All‐For‐One Vet Kit (Indical Biosciences) on the KingFisher Flex purification system (ThermoFisher, UK) and eluted in 100‐μL nuclease‐free water. Extract was stored at −80°C prior to RT‐PCR analysis.

The FTA™ cards were cut into four equally sized pieces (approximately 1 cm^2^) and placed into Precellys tubes. Then 500‐μL AVL buffer (Qiagen, UK) was added to each tube and the samples homogenised as described above. An equal volume of 500‐μL molecular grade 99.8% ethanol (Fisher Scientific) was added to the homogenate and the samples mixed by inversion. Samples were centrifuged at 13,000 rpm for 2 min to pelletise the card; thereafter, the supernatant was transferred to a QIAshredder tube and centrifuged at 13,000 rpm for 120 s. Total RNA was extracted using the QIAamp Viral RNA minikit (Qiagen, UK) and eluted in 80‐μL nuclease‐free water. The extract was stored at −80°C prior to RT‐PCR analysis.

The RT‐PCR primers and probe set were developed targeting a 61 bp fragment sequence in the NS5 region of WNV (Hadfield et al., 2001). Samples were analysed using the TaqMan Fast Virus Step‐1 Master Mix (ThermoFisher) on the QuantStudio 7 Flex Real‐Time PCR platform using the following run conditions: reverse transcription 50°C for 5 min, denature at 95°C for 20 s, followed by 45 cycles at 95°C for 3 s and 60°C for 30 s with a cool step of 40°C for 30 s. WNV strain NY99 was used as a positive RNA control alongside non‐template negative controls.

### Statistical analysis

All statistical analyses were performed in R (version 4.4.1) (R Core Team, 2021). The total adult female mosquito numbers for each trapping period are reported as the total number per trapping period (seven nights) and shown by trap type and wetland site. To investigate species abundance and composition between BGS and MM traps, all Box‐Gravid trap data were excluded from further analyses. To investigate the effect of trap type on the diversity of captured mosquitoes over the entire trapping period, species richness and Simpsons diversity index (1 − *D*) were calculated for each trap type, inclusive of all wetlands and for each wetland separately, using the *Vegan* package (Oksanen, 2015).

To assess mosquito abundance patterns across trap type, wetland and species, a zero‐inflated negative binomial (ZINB) model using the full dataset was constructed with trap location included as a random effect to account for repeated measures (Barton, 2024; Bates, 2014). Model selection was informed by Akaike information criterion (AIC), residual inspection and assessment of overdispersion (Zeileis et al., 2008; Zuur et al., 2009). Collinearity was tested between explanatory variables using variance inflation factor (VIF) and model specification and diagnostics were evaluated using the *DHARMa* package (Hartig, 2022). Models were selected based on improved model fit via likelihood ratio tests (analysis of deviance, ANODEV) and lower AIC scores. Post hoc testing was performed using Tukey's test to compare mean mosquito catch rates by wetland, with Holm's *p* value adjustment.

Based on the optimal structure of the full model, the same model structure (excluding the species term) was applied to species‐specific subsets to examine individual responses to trap and wetland. This nested approach is supported by the ‘top‐down’ strategy described in Zuur et al. (2009), which recommends fitting a comprehensive model to identify the optimal random and fixed structures before applying the same model logic to subgroups (Zuur et al., 2009).

Where data allowed, the species‐level models provided meaningful estimates of the effect of trap type and wetland on abundance. In cases with sparse data or zero counts, model convergence issues or wide confidence intervals limited analysis.

## RESULTS

**FIGURE 2:**
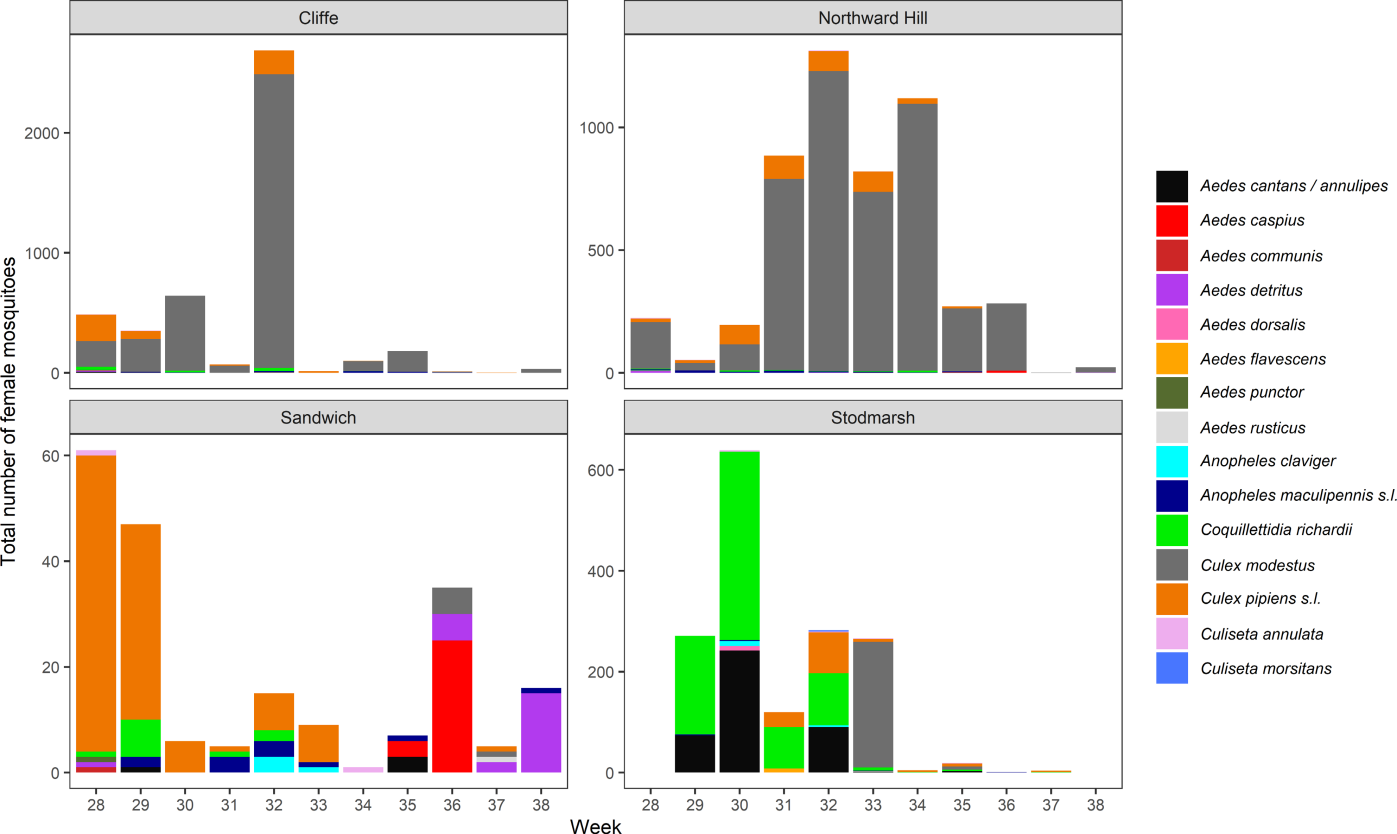
Numbers of adult female mosquitoes caught in all three trap types (Mosquito Magnet® Executive [MM], Biogents BG‐Sentinel [BGS], Box Gravid) at four wetlands (Cliffe, Northward Hill, Sandwich, Stodmarsh) shown by calendar week.

Three trap types at four wetlands ran from week 27 to 38 (4 July–23 Sept) and trapped a total of 11,584 adult female mosquitoes. At Northward Hill and Cliffe the highest number of mosquitoes were trapped (5,187 and 4,583 respectively), followed by 1,607 at Stodmarsh and 207 at Sandwich (Table 1). Overall across all wetlands, 15 species were recorded, the most abundant being *Cx. modestus*, followed by *Cx. pipiens s.l*., then *Coquillettidia richiardii*. The highest weekly catches of mosquitoes were observed in week 32 (8 August) at both Cliffe (2,686♀; 383.7♀/Trap night [TN]) and Northward Hill (1,313♀; 187.6♀/TN), dominated by *Cx. modestus* at both wetlands, while the highest numbers were observed at Stodmarsh in week 30 (25 July; 639♀) dominated by *Ae. cantans* /*annulipes* and *Cq. richiardii*. Overall abundances were much lower at Sandwich, with highest numbers of mosquitoes caught in weeks 28/29 (11 July [60♀]; 18 July [47♀]) dominated by *Cx. pipiens s.l*. (Figure 2).

**TABLE 1:** Numbers of adult female mosquitoes caught in all three trap types (Mosquito Magnet® Executive [MM], Biogents BG‐Sentinel [BGS], Box Gravid [BG]) at four wetlands (Cliffe, Northward Hill, Sandwich Bay and Stodmarsh).

Species	Cliffe				Northward Hill				Sandwich				Stodmarsh				Total
	MM	BGS	BG	Total	MM	BGS	BG	Total	MM	BGS	BG	Total	MM	BGS	BG	Total	
*Aedes cantans* /*annulipes*	10	1	0	11	2	1	0	3	4	0	0	4	372	39	0	411	**429**
*Aedes* (*Ochlerotatus*) *caspius* (Pallas 1771)	0	0	0	0	10	0	0	10	28	0	0	28	0	0	0	0	**38**
*Aedes* (*Ochlerotatus*) *communis* (de Geer, 1776)	0	0	0	0	0	0	0	0	0	1	0	1	0	0	0	0	**1**
*Aedes* (*Ochlerotatus*) *detritus* (Haliday, 1833)	10	0	0	10	9	1	0	10	22	1	0	23	0	0	0	0	**43**
*Aedes* (*Ochlerotatus*) *dorsalis* (Meigen, 1830)	0	0	0	0	0	0	0	0	0	0	0	0	9	0	0	9	**9**
*Aedes* (*Ochlerotatus*) *flavescens* (Muller, 1764)	5	0	0	5	2	0	0	2	0	0	0	0	0	8	0	8	**15**
*Aedes* (*Ochlerotatus*) *punctor* (Kirby, 1837)	0	0	0	0	0	0	0	0	0	1	0	1	0	0	0	0	**1**
*Aedes* (*Ochlerotatus*) *rusticus* (Rossi, 1790)	0	0	0	0	0	0	0	0	1	0	0	1	1	0	0	1	**2**
*Anopheles* (*Ano*.) *claviger* (Meigen, 1804)	1	0	0	1	0	1	0	1	3	1	0	4	14	0	0	14	**20**
*Anopheles* (*Ano*.) *maculipennis s.l*.	31	13	7	51	25	7	11	43	8	0	3	11	4	2	0	6	**111**
*Coquillettidia* (*Cq*.) *richiardii* (Ficalbi, 1889)	42	23	0	65	10	12	0	22	10	0	1	11	490	271	3	764	**862**
*Culex* (*Bar*.) *modestus* Ficalbi 1890	3180	738	0	3918	3118	1576	0	4694	5	1	0	6	6	249		255	**8873**
*Culex pipiens s.l*.	0	475	41	516	18	371	5	394	7	13	95	115	6	122	2	130	**1155**
*Culiseta* (*Culiseta*) *annulata* (Schrank, 1776)	2	1	2	5	2	5	1	8	1	0	1	2	6	1	0	7	**22**
*Culiseta* (*Culicella*) *morsitans* (Theobald, 1901)	0	1	0	1	0	0	0	0	0	0	0	0	0	2	0	2	**3**
Total	3281	1252	50	4583	3196	1974	17	5187	89	18	100	207	908	694	5	1607	**11,584**

In total 11,584 adult female mosquitoes and 24 FTA™ cards tested negative for WNV.

In total across all wetlands, four species (*Anopheles maculipennis s.l*. [21♀], *Cq. richiardii* [4♀], *Culiseta annulata* [4♀], *Cx. pipiens s.l*. [143♀]) were recorded at the Box‐Gravid trap. The majority of adult female mosquitoes in this trap were trapped at Sandwich (100 ♀), where *Cx. pipiens s.l*. represented 95% of the total catch.

**FIGURE 3:**
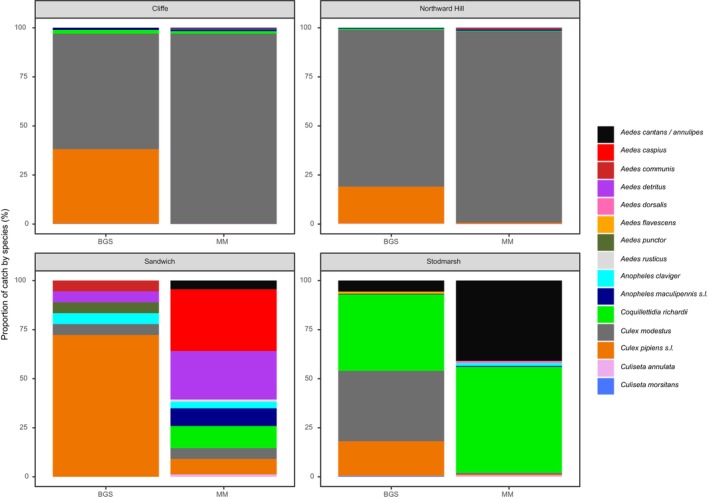
Proportion of adult female mosquitoes caught across all trap weeks, at two traps (Biogents BG‐Sentinel [BGS] and Mosquito Magnet® Executive [MM]) at four wetlands.

Comparing the MM and BGS traps, the MM traps caught a higher number of adult female mosquitoes per trap week (total = 7,474♀; mean = 70.5; SE = 25.7) compared to the BGS traps (3,938♀; mean = 51.8; SE = 14.6). Broadly, *Cx. modestus* dominated the catch in both trap types at Cliffe (86.4%) and Northward Hill (90.8%), whilst at Sandwich, *Cx. pipiens s.l*. dominated the BGS catch (76.5%), whilst a range of species were represented in the catch at the MM, particularly *Aedes caspius* (Figure 3). *Culex modestus* (35.9%) and *Cq. richiardii* (39.0%) together represented 74.9% of the overall catch at the BGS in Stodmarsh, whilst *Ae. cantans* (40.9%) and *Cq. richiardii* (54.0%) dominated at the MM (94.9% combined). Both trap types caught 12 species each over the entire sampling period, with the highest combined number of species recorded at Sandwich. Across all wetlands, the MM consistently caught a greater number of species; however, the Simpson's diversity index was greater for the BGS at three out of four wetlands, and greatest overall across all wetlands (Table 2). This suggests that whilst the MM caught greater total numbers of mosquitoes, its catches were proportionally dominated by few species and the BGS sampling represented greater species evenness.

**TABLE 2:** Species richness and species diversity indices (Simpson's index) shown for Mosquito Magnet® (MM) and BG‐Sentinel (BGS) at four wetlands (Cliffe, Northward Hill, Sandwich, Stodmarsh).

	Total number of species	Species richness		Species diversity (Simpson's index [1 − *D*])	
		BGS	MM	BGS	MM
Cliffe	10	7	8	0.508	0.060
Northward Hill	10	8	9	0.327	0.048
Sandwich	12	6	10	0.463	0.806
Stodmarsh	11	8	9	0.685	0.540
Overall	**15**	**12**	**12**	**0.508**	**0.279**

**FIGURE 4:**
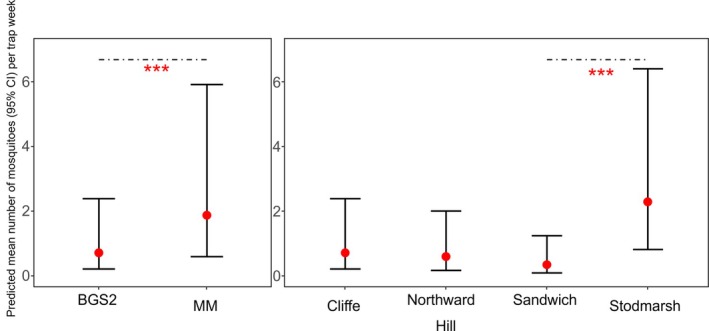
Predicted number of adult mosquitoes shown with 95% CI, trapped by trap type (Biogents BG‐Sentinel [BGS]; Mosquito Magnet® Executive [MM]) and wetland (Sandwich, Cliffe, Northward Hill, Stodmarsh), across all trap weeks. Predicted mean number per trap week from the selected model is shown (red circle) with bars representing 95% CIs. Significance (p < 0.001) is indicated by ‘***’.

Model selection indicated that the best‐fitting model for explaining variation in mosquito catch per trap night was the zero‐inflated negative binomial model including wetland, trap type and species as fixed effects, and trap location as a random effect. This model had the lowest AIC (2091.4) compared to all other candidate models, including those omitting one or more predictor variables (Table 3). Inclusion of all three variables significantly improved model fit relative to simpler models (ΔAIC ≥ 8.6). To evaluate the contribution of each predictor variable in the final model (Table 4), likelihood ratio tests were performed (ANODEV). Removal of wetland as a predictor resulted in a modest increase in AIC (from 2091.4 to 2103.7) and a significant decrease in model fit (*χ* ^2^ = 18.26, df = 3, *p* = 0.0004). Removal of the species variable had a larger impact, increasing the AIC to 2321.2 and reducing model fit significantly (*χ* ^2^ = 257.8, df = 14, *p* < 0.0001). Likewise, omitting trap as a variable led to an increase in AIC to 2321.8, and a significant reduction in model fit (*χ* ^2^ = 260.4, df = 15, *p* < 0.0001; Figure 4). These results confirm that all three variables of wetland, trap type and species contributed significantly to explaining variation in mosquito abundance, justifying their inclusion in the final model. Post hoc testing revealed that the mean number of adult female mosquitoes caught per trap week at Sandwich was significantly lower than at Stodmarsh (*p* = 0.0003; Figure 4), while differences between Sandwich and other wetlands (Cliffe and Northward Hill) were not statistically significant after adjustment (*p* > 0.1).

**TABLE 3:** Model variables: 1 = Wetland (Cliffe; Northward Hill; Sandwich; Stodmarsh); 2 = Trap (Biogents BG‐Sentinel [BGS]; Mosquito Magnet® Executive [MM]); 3 = Species.

Model variables	df	logLik	AIC	ΔAIC
1 + 2 + 3^a^	18	−1023.7	−1023.7	2091.4
1 + 3	17	−1029	−1029	2100
2 + 3	15	−1032.8	−1032.8	2103.7
3	14	−1037.4	−1037.4	2110.9
1 + 2	4	−1152.6	−1152.6	2321.2
1	3	−1153.9	−1153.9	2321.8
2	1	−1178.2	−1178.2	2366.5

*Note*: A random effect of trap location was also included.

Abbreviation: AIC, Akaike information criterion.

^a^ Indicates selected model.

**TABLE 4:** Outputs from the selected generalised linear effect model explaining the effects of wetland, species and trap type on the number of adult female mosquitoes.

	Estimate	Std. error	*z* Value	Pr (>|*z*|)
Conditional model				
(Intercept)	−0.344	0.620	−0.553	0.580
Trap type (baseline: BGS)				
MM	0.977	0.297	3.303	0.001
Wetland (baseline: Cliffe)				
Northward Hill	−0.174	0.349	−0.499	0.618
Sandwich	−0.795	0.442	−1.662	0.097
Stodmarsh	1.175	0.420	2.796	0.005
Species (baseline: *Ae. cantans* /*annulipes*)				
*Aedes caspius*	−0.958	0.659	−1.455	0.146
*Aedes communis*	−4.703	1.210	−3.888	0.000
*Aedes detritus*	−0.860	0.646	−1.331	0.183
*Aedes dorsalis*	−3.478	0.701	−4.964	0.000
*Aedes flavescens*	−2.395	0.646	−3.705	0.000
*Aedes punctor*	−4.703	1.210	−3.888	0.000
*Aedes rusticus*	−4.161	0.958	−4.343	0.000
*Anopheles claviger*	−2.256	0.659	−3.426	0.001
*Anopheles maculipennis s.l*.	0.036	0.632	0.057	0.955
*Coquillettidia richiardii*	1.238	0.562	2.203	0.028
*Culex modestus*	4.302	0.633	6.792	0.000
*Culex pipiens s.l*.	3.024	0.655	4.615	0.000
*Culiseta annulata*	−1.750	0.674	−2.595	0.009
*Culiseta morsitans*	−3.552	0.852	−4.167	0.000

Abbreviations: BGS, Biogents BG‐Sentinel; MM, Mosquito Magnet® Executive.

**FIGURE 5:**
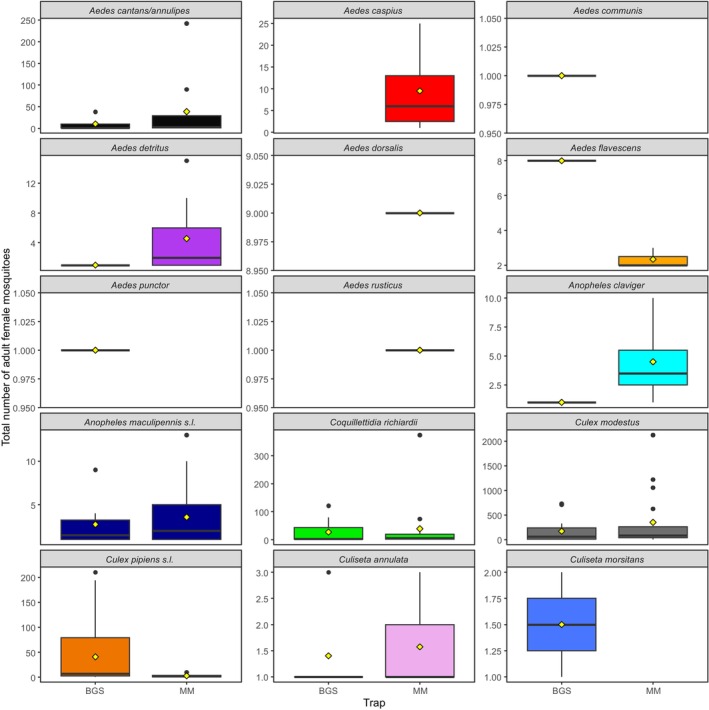
Boxplots showing the total number of each species caught per trap week across all wetlands by trap type (Biogents BG‐Sentinel [BGS] and Mosquito Magnet® Executive [MM]). Boxplots show the inter quartile range (Q1 and Q3) below and above the median line. Means are shown as yellow diamonds; outliers are represented by black circles.

**FIGURE 6:**
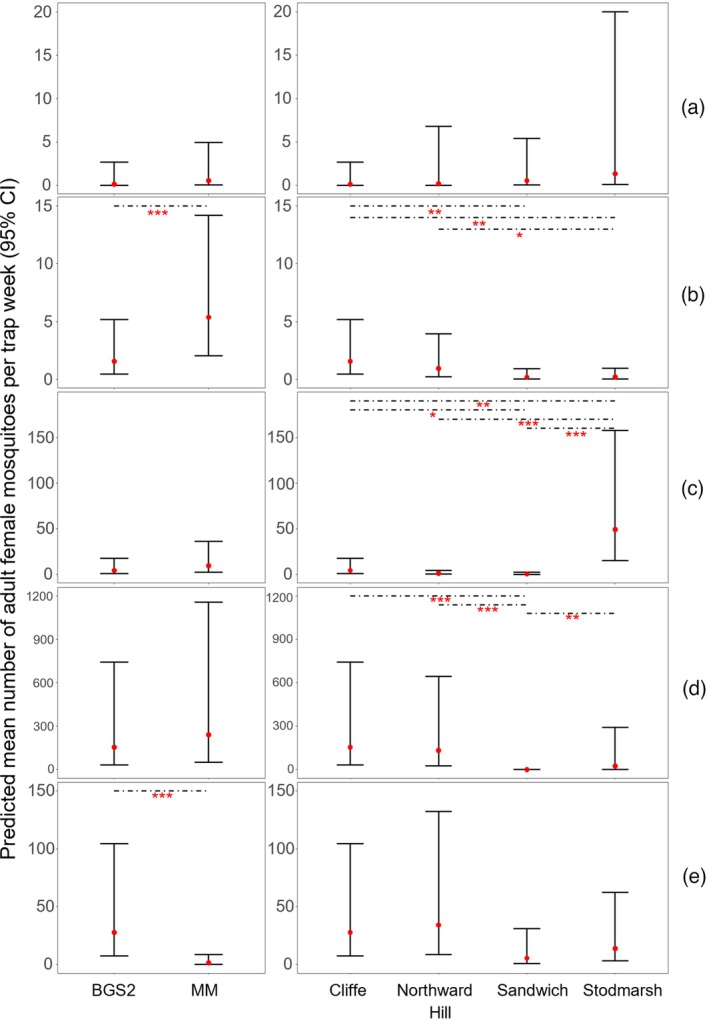
Predicted mean number (red circle) of adult female mosquitoes per trap week, with bars representing 95% confidence intervals (CIs), shown by trap type (Biogents BG‐Sentinel [BGS] and Mosquito Magnet® Executive [MM]) and wetland (Cliffe, Northward Hill, Sandwich, Stodmarsh). Rows represent species: A = *Anopheles claviger*; B = *Anopheles maculipennis s.l*.; C = *Coquillettidia richiardii*; D = *Culex modestus*; E = *Culex pipiens s.l*. Significant comparisons as determined by post hoc tests are indicated by dashed line and the asterix symbol (*** = p ≤ 0.001; ** = 0.001 < p ≤ 0.01; * = 0.01 < p ≤ 0.05. ). Remaining species models would not converge and therefore predicted means and CIs could not be estimated.

Species‐specific models revealed heterogeneous responses to trap type and wetland, despite the overall dominance of the MM trap in total mosquito abundance (Table 5). While MM traps consistently collected more mosquitoes overall, the effect of trap type was not uniform across species (Figure 5). *Culex pipiens s.l*. was significantly more abundant in BGS traps (*p* < 0.001), in contrast to *An. maculipennis s.l*., which had a significantly higher abundance at the MM traps (*p* = 0.01; Figure 6). Conversely, *Cx. modestus*, *Cq. richiardii* and *Cs. annulata* did not show statistically significant differences between trap types. Several species, including *Ae. flavescens*, *Ae. dorsalis* and *Ae. detritus*, could not be assessed due to convergence issues, likely related to sparse or highly clustered count data. Where interpretable, the effects of the wetland variable were significant for some species, especially for *Cq. richiardii*, which was more abundant at Stodmarsh and Sandwich, and *Cx. modestus*, which showed a marked reduction in catch rates at Sandwich compared to all other wetlands.

**TABLE 5:** Estimated trap type and wetland effects on mosquito abundance for species, based on species‐specific zero‐inflated negative binomial models.

	MM		Northward		Sandwich		Stodmarsh	
	*ꞵ*	*p*	*ꞵ*	*p*	*ꞵ*	*p*	*ꞵ*	*p*
*Anopheles claviger*	1.620	0.224	0.600	0.780	1.660	0.194	2.570	0.030
*Anopheles maculipennis s.l*.	1.230	0.011	−0.500	0.354	−2.080	0.001	−2.000	0.003
*Coquillettidia richiardii*	0.790	0.183	−1.220	0.113	−2.270	0.007	2.410	0.001
*Culiseta annulata*	0.470	0.427	0.610	0.456	−1.350	0.274	0.770	0.352
*Culex modestus*	0.440	0.449	−0.160	0.796	−6.180	<0.001	−1.840	0.141
*Culex pipiens s.l*.	−2.970	<0.001	0.210	0.778	−1.610	0.080	−0.690	0.378

*Note*: Values represent model estimates (*β*) and associated *p*‐values. The baseline category is the BGS trap at Cliffe wetland for the species *Aedes cantans* /*annulipes*. Significant effects (*p* < 0.05) are indicative of differences in catch rate relative to the baseline. Remaining species could not be estimated due to insufficient data.

Abbreviations: BGS, Biogents BG‐Sentinel; MM, Mosquito Magnet® Executive.

## DISCUSSION

This study, conducted over 12 weeks across four wetlands in Kent, provides insights into how two widely utilised mosquito traps, the BG‐Sentinel and the Mosquito Magnet®, differ in trapping efficiency, reflected by differences in captured species diversity and abundance. Overall, data demonstrated significant differences in mean capture rates between the two trap types, where the total number of mosquitoes captured by MMs was significantly greater than BGS. Interestingly, despite trapping lower overall numbers of mosquitoes, the BGS traps captured a greater diversity of species. In addition, a Box‐Gravid trap was tested at each wetland, and an FTA™ card system was utilised in order to test for West Nile in mosquito saliva, as well as testing mosquitoes trapped in all three trap types. A total of 11,584 adult female mosquitoes were captured, with Northward Hill and Cliffe having the highest densities, capturing 5,187 and 4,583 mosquitoes, respectively. These high captures compared to the notably lower counts at Stodmarsh (1,607) and Sandwich (207) suggest substantial spatial variation in mosquito populations across these wetlands, likely driven by species‐specific larval habitat availability.

A total of 15 species were recorded overall. *Cx. modestus* was the most prevalent, followed by *Cx. pipiens s.l*. and *Cq. richiardii*, with peaks in abundance recorded in early August at Cliffe and Northward Hill, dominated by *Cx. modestus*. Notably, the dominant species by proportion of total captured mosquitoes varied by wetland and sample week, with *Ae. cantans* /*annulipes* and *Cq. richiardii* dominating at Stodmarsh in late July, and *Ae. caspius* dominating at Sandwich in early September. This pattern emphasises the importance of wetland‐specific factors which influence mosquito community composition, and the conclusions which can be drawn from such studies. Cliffe and Northward Hill's high captures likely reflect suitable larval conditions and host availability, with permanent, vegetated, shallow ditches and grazing livestock favourable for supporting *Cx. modestus* populations. This is well documented in the existing literature and aligns with the species' common exploitation of permanent ditch habitats in grazing marshes, which are highly abundant in these two locations (Medlock & Vaux, 2012; Vaux et al., 2015, 2024). *Culex modestus* was also present at Stodmarsh, although densities were considerably lower compared to Cliffe and Northward Hill, perhaps reflective of where the traps were situated, in close proximity to a mosaic of wetland habitat including wet woodland and reedbed, and some distance from permanent ditch habitat more suited to this species. Low numbers of *Cx. modestus* were also found at Sandwich, with this study recording this species' presence for the first time, despite continuous surveillance efforts using a Mosquito Magnet®, since 2010, providing evidence for a geographic expansion of this species into this area (Vaux et al., 2024). Habitat variation across the wetlands is reflected in the relatively low abundances of mosquitoes across all species at Sandwich, although species richness was highest here (12), compared to other wetlands (Northward Hill & Cliffe = 10; Stodmarsh = 11); however, this study has not quantified the scale of available habitat at each wetland. Future studies should seek to consider this to inform analyses which may give a more accurate appraisal of the relative trapping efficiency and species‐specificity of these two widely used trap types.

The MM and BGS traps performed differently in this study overall. Mosquito Magnet® traps captured a significantly greater total number of adult females across all species and wetlands (7,474 females; mean per trap week = 70.5) compared to BGS traps (3,938 females; mean per trap week = 51.8). Both traps caught an equal number of species; however, some species were represented in only one trap type. Overall, the selected model to examine variation in mosquito abundance per trap week indicated that wetland, species and trap type significantly influenced mosquito abundance. Further post hoc testing revealed that Sandwich was significantly different from the other wetlands, further emphasising that the wetland has unique environmental characteristics relevant to mosquito ecology compared to the others.

This trend of higher captures by the MM trap suggests it is more effective at attracting mosquitoes from across a wider area, possibly due to its design characteristics that utilise heat and water vapour, which BGS traps do not, and greater rates of CO_2_ production, as compared to the BGS. The BGS CO_2_ system was set to output 175 mL/min. Whilst the average flow rate of the MM is not published by the manufacturer in these terms, taken from the manufacturer's website, a 20 lb (9.07 kg) cylinder of propane will last approximately 21 days at constant use (Woodstream, 2022). A 20 lb. cylinder contains approximately 17.44 L of propane. Using a stoichiometric equation (C_3_H_8_ + 5O_2_ → 3CO_2_ + 4H_2_O), it can be calculated that an estimated 25.73 kg of carbon dioxide is produced when the propane is combusted over 21 days. Assuming that the production of CO_2_ is constant across this operational period, this would be a flow rate of approximately 462.96 mL/min (51.06 g/h; 0.85 g/min), over 2.5 times the rate at the BGS. Higher rates of CO_2_ release have been shown to significantly increase the number of mosquitoes caught in traps, particularly mammalophilic species (McPhatter & Gerry, 2017; Mullens & Gerry, 1998), which may have influenced the results here, although the effect of different lure types cannot be ruled out. Despite higher numbers of mosquitoes trapped at the MM overall, species richness and diversity indices highlighted interesting contrasts: while both traps captured 12 species each, the BGS trap had a higher Simpson index (1 − *D*) at three of the four wetlands, indicating greater evenness in the relative abundance of different species captured, compared to the MM traps, rather than the predominance of one or a few species. This implies that the BGS is less species‐selective and may attract a more diverse range of species in comparable proportions, whereas the MM may disproportionately attract specific mosquito species in higher numbers.

Species‐level modelling revealed significant differences across trap type and wetland. *Anopheles maculipennis s.l*. was captured at significantly higher rates at the MM compared to the BGS, but there was no significant difference between wetlands. *Coquillettidia richiardii* exhibited no difference in abundance by trap type, but was significantly higher at Stodmarsh when compared to the other wetlands, likely driven by the presence of large reedbed and emergent vegetation habitats at that wetland. This species utilises an adaptation whereby larvae draw oxygen from the submerged plants in these habitats and therefore large expanses of these habitats would likely support large populations of this species. *Culex modestus* was recorded at both the MM and BGS, with no significant difference between the two trap types; though significant wetland‐level differences were seen for this species, with Sandwich being significantly different to each of the other wetlands, with only 6 adults caught at Sandwich. *Culex pipiens s.l*. was trapped in significantly greater numbers at the BGS compared to the MM, and no difference was seen at the wetland level. No significant differences were seen across trap type or wetland for *Anopheles claviger*. Analyses could not elucidate the effect of trap type or wetland on other species recorded in this study. *Aedes cantans* /*annulipes* was recorded at both trap types in relatively large numbers (Figure 5). Remaining species (*Ae. caspius*, *Ae. communis*, *Ae. detritus*, *Ae. dorsalis*, *Ae. flavescens*, *Ae. punctor*, *Ae. rusticus*, *Cs. annulata* and *Cs. morsitans*) were recorded in too low numbers for a statistical comparison, likely to be a result of their low density at these wetlands.

The findings that trap type was a significant predictor of abundance for *Cx. pipiens s.l*. and *An. maculipennis s.l*. suggests that these species have inherent preferences which are exploited by certain features or attractants present at each trap type, likely a result of olfactory differences including type of lure, rate of CO_2_ emission, heat outputs or physical trap characteristics such as size, shape or entry point. This study did not seek to assess these characteristics, but sought to draw practical conclusions as to the use of these host‐seeking adult mosquito traps in surveillance projects in UK wetlands.

This study trialled the use of a Box‐Gravid trap for the first time in a wetland setting in the United Kingdom, although they have been used to target *Cx. pipiens s.l*. in the United Kingdom in residential gardens (Townroe & Callaghan, 2015). The Box‐Gravid trap captured lower numbers of mosquitoes compared to the other two trap types overall and captured only four species, compared to the 12 at both other traps, although the Box‐Gravid traps were not rotated, potentially limiting collection efficacy compared to the other two traps. This, however, is not unexpected, given that this type of trap is likely to attract only species that oviposit in container habitats, such as *Cx. pipiens s.l*. and *Cs. annulata*, as well as species that exploit permanent water bodies such as *An. maculipennis s.l*. and *Cq. richiardii*. A similar study in Germany, utilising a Box‐Gravid trap fitted with an FTA™ card, also recorded *Cx. pipiens s.l*. as the most abundant species, as well as *Cs. annulata* and *An. maculipennis s.l*. (Fynmore et al., 2021). In this study, 172 adult female mosquitoes were trapped at the Box‐Gravid traps, with the majority of these (58%) caught at Sandwich. The effectiveness of this type of trap is likely related to the availability of aquatic habitats, as well as the propensity of the mosquito species present to oviposit in an artificial container habitat, with hay‐infused water. Improvements to the trap may increase catch rate, such as an increase in fan speed and therefore air currents over the water, and maintenance of the water level within ~1–5 cm of the aperture into the trap to ensure optimal airflow, although the latter is influenced by evaporation rate.

Analyses for WNV found no evidence of virus circulation in either the mosquitoes or the FTA™ cards. The recent detection of WNV in *Ae. vexans* at a wetland in Nottinghamshire in 2023 has shown the potential for WNV in a UK landscape, and also the value of utilising species not considered to be primary vectors as biosentinels (Bruce et al., 2025). Use of FTA™ cards has been proven to be an effective tool in numerous studies, including in European wetland settings (Birnberg et al., 2020; Fynmore et al., 2021; Hall‐Mendelin et al., 2010; Manzi et al., 2023; Wipf et al., 2019), and they were therefore utilised in this study conducted in an area with known WNV vector species present. However, their feasibility for viral surveillance in the United Kingdom should be the subject of future study. Enhanced mosquito capture rates at an FTA™ augmented trap would increase sugar feeding rates at the FTA™ card and therefore provide greater probabilities of virus detection. Although the Box‐Gravid trap selects for mosquitoes that have previously taken a bloodmeal, the use of FTA™ cards in traps designed to exploit host‐seeking behaviour (Manzi et al., 2023) may increase virus detection rates, whilst the usage of attractive sugar bait traps may provide another improved alternative method (Lothrop et al., 2014). Although it should be noted that WNV can be transmitted transovarially and therefore unfed mosquitoes may still be infected with the virus, meaning that trap‐based surveillance efforts need not exclusively target blood‐fed mosquitoes (Anderson et al., 2014; Baqar et al., 1993; Dohm et al., 2002; Nelms et al., 2013).

This study's findings highlight considerable variation in mosquito abundance across wetlands, with Northward Hill and Cliffe being particularly productive for mosquito captures. No data were collected or analysed on weather to investigate differences across site, although observations in the field noted consistently windy conditions in the more exposed wetlands of Cliffe and Northward Hill. The MM trap captured significantly more mosquitoes overall, yet the BGS trap demonstrated higher diversity indices and a greater effectiveness in capturing *Cx. pipiens s.l*., an important enzootic vector. *Culex modestus*, considered a primary WNV vector in Europe (Balenghien et al., 2007; Mouchet et al., 1970; Ponçon et al., 2007), displayed no difference in abundance by trap type. Species trends were determined by trap type (e.g., *Cx. pipiens s.l*.) or wetland (e.g., *Cq. richiardii*), whilst others showed no difference across either (e.g., *An. claviger*), indicating that surveillance and research studies targeting particular species need to establish the optimal trap and habitat specific to the species in order to maximise catch rates. For mammalophagic mosquito monitoring, the MM trap may be beneficial due to higher total captures, while the BGS trap appears more suited to studies focusing on species diversity or to specifically target *Cx. pipiens s.l*. populations. However, both traps have their own operational requirements, such as provision of propane gas or access to sunlight for the photovoltaic panels, and it is these requirements that often influence decisions around trap type. Data presented here can guide targeted vector surveillance, particularly in studies of British species where specific species are of concern for disease transmission. However opportunities to develop data on species trap rates should be included in future studies, particularly targeting species underrepresented in this study, such as human‐biting *Ae. caspius* and *Ae. detritus*. Based on the results of this study, the BGS trap should be used to target a wider range of species, importantly also including ornithophagic species like *Cx. pipiens s.l*., in order to detect circulation of pathogens within enzootic transmission cycles. When assessing risk of human‐biting species, and where studies aim to target mammalophagic species, the MM should be used to ensure these species are represented in trapping samples and provide data on potential for human biting. Overall, these data confirm that MM and BGS traps operate differently in the United Kingdom, specifically in the numbers of mosquitoes they capture, targeted species, and the relative proportions of captured species. This has important consequences when considering both species‐specific surveillance and the design of studies which seek to estimate mosquito population densities.

## AUTHOR CONTRIBUTIONS

**Alexander G. C. Vaux:** Conceptualization; project administration; methodology; investigation; writing – original draft; data curation; formal analysis; visualization. **Harrison Hardy:** Writing – review and editing; formal analysis. **Lucy Crossley:** Methodology; investigation; writing – review and editing. **Colin J. Johnston:** Investigation; conceptualization; writing – review and editing. **Anthony J. Abbott:** Investigation; writing – review and editing. **Stephen Findlay‐Wilson:** Methodology; investigation; writing – review and editing. **Amanda Callaghan:** Writing – review and editing; methodology. **Jolyon M. Medlock:** Project administration; methodology; investigation; writing – review and editing.

## CONFLICT OF INTEREST STATEMENT

The authors declare no conflicts of interest.

## Supporting information


**Figure S1.** Map of the British Isles, showing the location of the four study sites.

## Data Availability

The dataset for this study is available at https://doi.org/10.5061/dryad.rbnzs7hrp (Vaux et al, 2025).
