# Supplementary figures and images for: Mosquito and arbovirus surveillance in wetlands of South‐East England: Comparison of two adult mosquito traps, use of a novel trap with FTA™ cards and arbovirus testing
Source: Med Vet Entomol. 2026 Feb 6;40(2):407–21. doi: 10.1111/mve.70053 (PMC13140037; doi:10.1111/mve.70053)

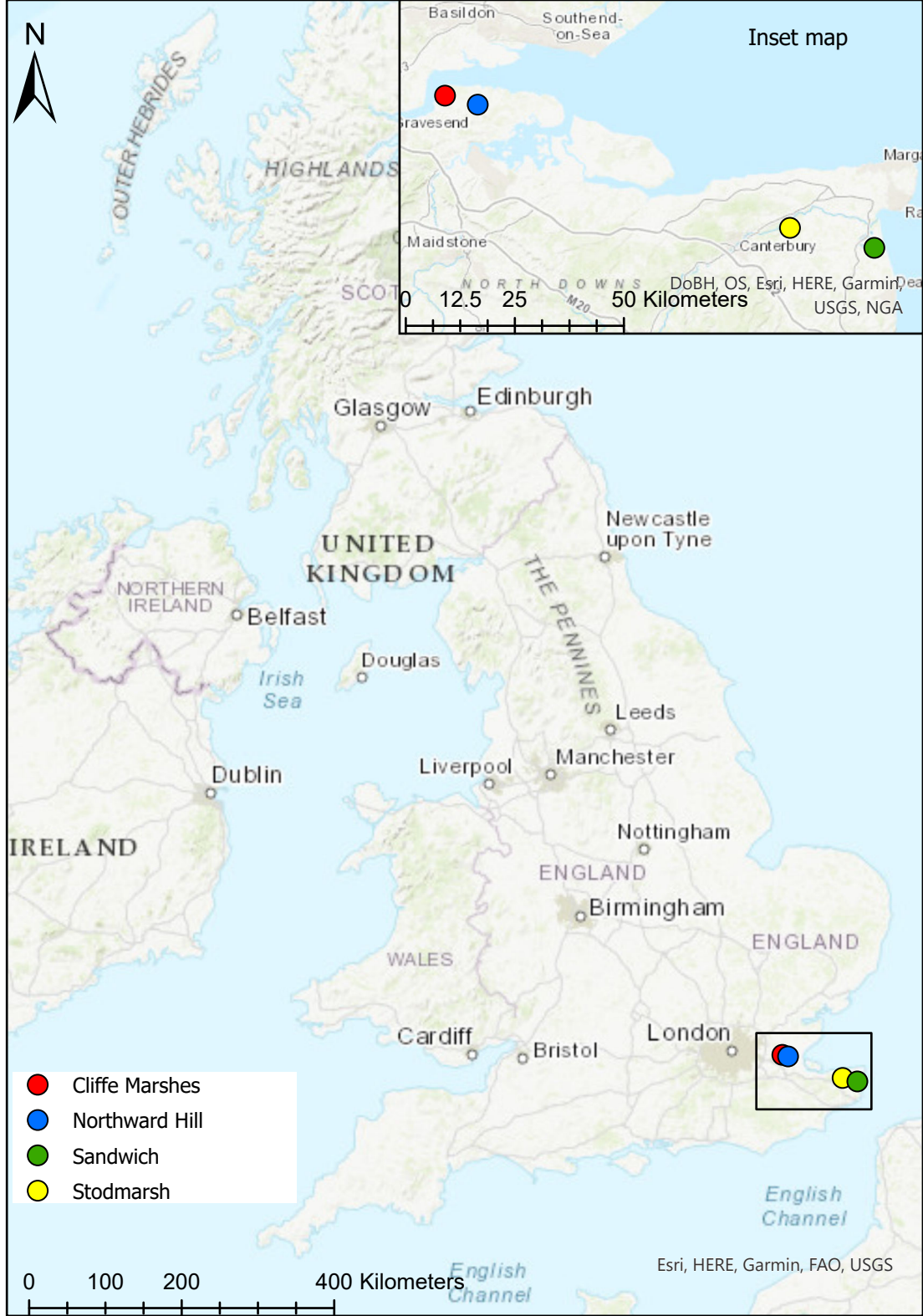

Supplement: Supplementary file 1 — Figure S1. Map of the British Isles, showing the location of the four study sites. [file MVE-40-407-s001.pdf]
